# SALVAGE SURGERY IN GASTRIC CANCER

**DOI:** 10.1590/0102-672020210002e1629

**Published:** 2022-01-31

**Authors:** Italo Beltrão Pereira SIMÕES, Marina Alessandra PEREIRA, Marcus Fernando Kodama Pertille RAMOS, Ulysses RIBEIRO, Bruno ZILBERSTEIN, Sergio Carlos NAHAS, Andre Roncon DIAS

**Affiliations:** 1 Trabalho realizado no Instituto do Câncer, Hospital de Clínicas - HCFMUSP, Universidade de São Paulo, Departamento de Gastroenterologia, Faculdade de Medicina da Universidade de São Paulo - USP - SP - São Paulo - Brasil

**Keywords:** Stomach Neoplasms, Gastrectomy, Endoscopic Surgical Procedures, Neoplasm Recurrence, Local, Neoplasias Gástricas, Gastrectomia, Procedimentos Cirúrgicos Endoscópicos, Recidiva Local de Neoplasia

## Abstract

Salvage surgery (SS) is defined as surgical resection after the failure of the first treatment with curative intent.

**AIM:**

The aim of this study was to report the experience of a reference center with SS for stomach adenocarcinoma.

**METHODS:**

This is a retrospective study of patients with gastric cancer (GC) operated on between 2009 and 2020.

**RESULTS:**

Notably, 40 patients were recommended for salvage gastrectomy with curative-intent treatment. For analysis purpose, patients were divided into two groups: 23 patients after endoscopic resection and 17 patients after gastrectomy. In the first group, all patients underwent R0 resection, their average hospital length of stay (LOS) was 15.7 days, and 2 (8.6%) patients had major complications. During the average follow-up of 37.2 months, there was only one recurrence. The median overall survival (OS) was 46 months. In the postgastrectomy group, 9 (52.9%) patients were rescued with curative intent, the average hospital LOS was 12.2 days, and 3 (17.6%) had major complications. In a mean follow-up of 22 months, five patients relapsed. Median OS and disease-free survival were 24 and 16.5 months, respectively.

**CONCLUSION:**

SS in GC offers the possibility of long-term disease control and increased survival rate with an acceptable complication rate.

## RESUMO - RACIONAL

A cirurgia de resgate é definida como a ressecção cirúrgica após falha de primeiro tratamento com intuito curativo.

**OBJETIVO:**

Relatar a experiência de um centro de referência no tratamento do câncer gástrico com a cirurgia de resgate para o adenocarcinoma de estômago.

**MÉTODOS:**

Análise retrospectiva dos pacientes com câncer gástrico operados entre 2009 e 2020.

**RESULTADOS:**

40 pacientes foram submetidos à tentativa de gastrectomia de resgate com intuito curativo. Para análise, foram divididos em dois grupos: 23 pacientes após ressecção endoscópica e 17 após gastrectomia. No primeiro grupo, todos tiveram ressecção com margens livres, a média de internação foi 15,7 dias e 2 (8,6%) tiveram complicações maiores. No seguimento médio de 37,2 meses, houve apenas 1 recidiva. A sobrevida global média foi 46 meses. No grupo pós-gastrectomia 9 (52,9%) foram resgatados com intenção curativa, a média de internação foi 12,2 dias e 3 (17,6%) apresentaram complicações maiores. No seguimento médio de 22 meses, 5 recidivaram. A sobrevida global média e a sobrevida livre de doença foram respectivamente: 24 e 16,5 meses.

**CONCLUSÃO:**

A cirurgia de resgate no câncer gástrico oferece nova possibilidade de controle da doença a longo prazo e/ou aumento de sobrevida, tendo taxa de complicações aceitáveis.

## INTRODUCTION

Gastric cancer (GC) is a common and highly lethal malignant neoplasm in which the curative-intent treatment involves resection.^23^ In early cases, when the tumor is restricted to the mucosa and submucosa without lymph node metastases, endoscopic resection is indicated. For all other cases, only surgical resection is potentially curative.^12^

Salvage surgery (SS) is performed when the patient has undergone a curative-intent treatment earlier, but the tumor persisted or recurred. Such concept emerged in the 1960s, referring to head and neck or gynecologic tumors that had been previously treated with definitive radiotherapy.^19^ ^,^ ^26^

There are three possible scenarios for SS in GC: after endoscopic resection, after regional recurrence, and after gastrectomy with a compromised surgical margin. The fourth anecdotal scenario would be lymphoma with exclusive gastric persistence after chemotherapy.

Nowadays, literature on SS for GC is extremely poor and it frequently has concept misplacements, considering palliative and conversion surgery as a synonym of SS.^6^ ^,^ ^27^ In addition, series are usually small and often include cases in whom the first procedure cannot be considered curative.^1^ It is worth mentioning that no studies are still available in Brazil on the subject.

## METHODS

All patients with gastric adenocarcinoma treated at our institution between 2009 and 2020 were considered. Our prospective database was reviewed, selecting the cases who underwent SS and then dividing them into three groups for analysis: after endoscopic resection, after regional recurrence, and after gastrectomy with compromised margins.

This study provided that the first treatment was performed with curative-intent method and followed the recommendations of the guidelines of the Japanese Gastric Cancer Association (JGCA).^12^ Salvage surgery is defined as a novel attempt to cure after persistence or relapse. Palliative patients were excluded. For the first group, the inclusion criterion was endoscopic resection. For the second group, the cases of exclusive regional recurrence located in the gastric stump, in the anastomoses, extraluminal in the previously dissected area, and/or in the regional lymph nodes (para-aortic lymph nodes were considered distant recurrence) were considered. The third group consisted of patients with positive margins after radical surgery. Cases previously treated at other institutions were also included. Patients undergoing inadequate lymphadenectomy or gastrectomy for suspected benign disease were excluded.

Postoperative follow-up was performed in a quarterly manner during the first year and every 6 months in the following years. Imaging exams for recurrent detection were performed in the presence of symptoms or due to clinical suspicion. Surgical complications were classified according to Clavien-Dindo clasification^4^ and divided into minor and major (Clavien > II) groups. Deaths until 30 days from surgery or during postoperative hospitalization were considered surgical mortality.

This study was approved by the Hospital Ethics Committee and is registered online (Plataforma Brasil, CAAE: 45053121.1.0000.0068).

### Statistical Analysis

Data are described as a function of mean, median, standard deviation (±SD), minimum and maximum for quantitative variables, and frequency and tables for qualitative variables. The t-test was performed to differentiate the quantitative variables. The association between categorical variables was determined using Pearson’s chi-square test or Fisher’s exact test. Overall survival (OS) and disease-free survival (DFS) were estimated using the Kaplan-Meier method, and the differences in survival were assessed using the log-rank test. The Cox proportional hazards model was used to determine the risk factors associated with the outcome. A 95% confidence interval (95% CI) was used. Variables that reached significance in the univariate analysis were included in the multivariate model. The p-values <0.05 were considered significant. The SPSS version 20.0 statistical program (SPSS Inc., Chicago, IL, USA) was used for statistical analyses.

## RESULTS

During the evaluation period, 23 patients who underwent endoscopic mucosal resection (EMR) or endoscopic submucosal dissection (ESD) were surgically rescued. The interval between the endoscopic resection and surgery was 6 months. All patients were resected with curative-intent treatment, and the subtotal and total gastrectomies were performed in 12 and 11 cases, respectively. Minimally invasive access was the preferred method (56.5%). The mean tumor size was 2.7 cm, 13% had stage pT3-4, and 82.6% were pN0. Free margins were obtained in all cases and the average hospital length of stay (LOS) was 15.7 days. Two patients had major complications. In a median follow-up of 37.2 months, one patient relapsed.

Scenarios 2 and 3 were analyzed together (n=17). There was only one patient with positive margins following the first surgery. A subtotal gastrectomy was the first procedure in 70.0% of the cases. Considering 17 patients, 52.9% received curative resection. The minimally invasive access was used in 3 (17.6%) patients. The mean tumor size was 4 cm, 35.0% of patients were pT4, and 47.1% were stage IV. Free margins were obtained in 52.9% of cases, the average hospital LOS was 12.2 days, and three patients had major complications. In the mean follow-up of 22 months, 55.6% of patients resected with curative-intent relapsed.

The clinical, pathological, and surgical data of the patients are given in Tables 1, 2 and 3. There was one patient of persistent gastric lymphoma after chemotherapy, who was treated with laparoscopic partial gastrectomy and Billroth-I reconstruction. The patient is disease-free in the current 12-month follow-up.

**Table 1 -:** Clinical characteristics of cases undergoing SS.

Variables	Post-EMR/ESD group		Postgastrectomy group	
	n=23	%	n=17	%
Sex				
Female	11	47.8	9	52.9
Male	12	52.2	8	47.1
Age (years)				
Mean (SD)	65.3 (15.1)		61.9 (12.5)	
Min-max	42.5 - 89.4		36.3-77.4	
Body mass index (kg/m²)				
Mean (SD)	23.4 (5.2)		23.1 (4.5)	
Hemoglobin (g/dL)				
Mean (SD)	12.7 (1.7)		11.9 (1.3)	
Albumin (g/dL)				
Mean (SD)	4.1 (0.4)		4.1 (0.5)	
Charlson-Deyo comorbidity index (CCI)				
0	15	65.2	12	70.6
≥1	8	34.8	5	29.4
American Society of Anesthesiologists (ASA)				
II	14	60.9	13	76.5
III	9	39.1	4	23.5
Type of initial resection				
Endoscopic	23	100.0	0	0.0
Subtotal	0	0.0	12	70.6
Total	0	0.0	1	5.9
Degastrectomy	0	0.0	1	5.9
Gastrectomy (nonspecified)	0	0.0	3	17.6
Time interval for salvage (years)				
Mean (SD)	0.6 (0.6)		2.4 (1.6)	
Average (min-max)	0.3 (0-2.6)		2 (1-6)	
Surgery type-Salvage				
Curative	23	100.0	9	52.9
Palliative	0	0.0	4	23.5
Diagnostic	0	0.0	4	23.5
Salvage surgery performed				
Subtotal gastrectomy	12	52.2	0	0.0
Total gastrectomy	9	39.1	0	0.0
Gastric-remnant resection	2	8.7	10	58.8
Colectomy	0	0.0	3	17.6
Nonresected	0	0.0	4	23.5
Access				
Conventional	10	43.5	14	82.4
Laparoscopic/robotic	13	56.5	3	17.6
Lymphadenectomy				
D1	3	13.0	0	0.0
D2	17	73.9	4	23.5
Not applicable	3	13.0	13	76.5
Disease location				
Anastomosis	1	4.3	7	41.2
Distal	10	43,5	4	23.5
Medial	5	21.7	0	0.0
Proximal	5	21.7	3	17.6
Others	0	0.0	3	17.6
Not specified	2	8.7	0	0.0

**Table 2 -:** Pathological characteristics of cases undergoing SS.

Variables	Post-EMR/ESD group		Postgastrectomy group	
	n=23	%	n=17	%
Lauren classification				
Intestinal	17	73.9	5	29.4
Diffuse/mixed	3	13.0	11	64.7
Neuroendocrine adenocarcinoma	0	0.0	1	5.9
Nonadenocarcinoma	3	13.0	0	0.0
Differentiation degree				
G1/G2	18	78.3	5	29.4
G3	5	21.7	11	64.7
Not applicable	0	0.0	1	5.9
Lymphatic invasion				
Absent	18	78.3	5	29.4
Present	5	21.7	6	35.3
Not applicable	0	0.0	6	35.3
Venous invasion				
Absent	22	95.7	8	47.1
Present	1	4.3	3	17.6
Not applicable	0	0.0	6	35.3
Perineural invasion				
Absent	19	82.6	3	17.6
Present	4	17.4	8	47.1
Not applicable	0	0.0	6	35.3
Tumor size				
Mean (SD)	2.7 (1.7)		4 (1.7)	
Average (min-max)	1.9 (0.9-6.6)		3.6 (1.7-7.5)	
pT				
pTx	0	0.0	7	41.2
pT1	18	78.3	1	5.9
pT2	2	8.7	0	0.0
pT3	1	4.3	3	17.6
pT4	2	8.7	6	35.3
Lymph nodes				
Mean (SD)	31 (17)		15.4 (9.6)	0.0
pN				
pNx	0	0.0	7	41.2
pN0	19	82.6	4	23.5
pN1	2	8.7	2	11.8
pN3	2	8.7	4	23.5
pTNM				
I	20	87.0	1	5.9
II	1	4.3	3	17.6
III	2	8.7	5	29.4
IV	0	0.0	8	47.1

**Table 3 -:** Surgical results of patients undergoing SS.

Variables	Post-EMR/ESD group		Postgastrectomy group	
	n=23	%	n=17	%
Margins				
R0	23	100.0	9	52.9
R2	0	0.0	8	47.1
Length of stay (days)				
Mean (SD)	15.7 (14.2)		12.2 (11.3)	0.0
Median (IQR)	11 (7-17)		9 (5-12.5)	0.0
Postoperative complications				
0-II	21	91.3	14	82.4
III-V	2	8.7	3	17.6
Follow-up time (months)				
Mean (SD)	37.2 (24.5)		22.3 (32.4)	
Median	32.7		10	
Recurrence (only curative)				
No	22	95.7	4	44.4
Yes	1	4.3	5	55.6
*Noncurative	*0*		*8*	

**Figure 1 -:**
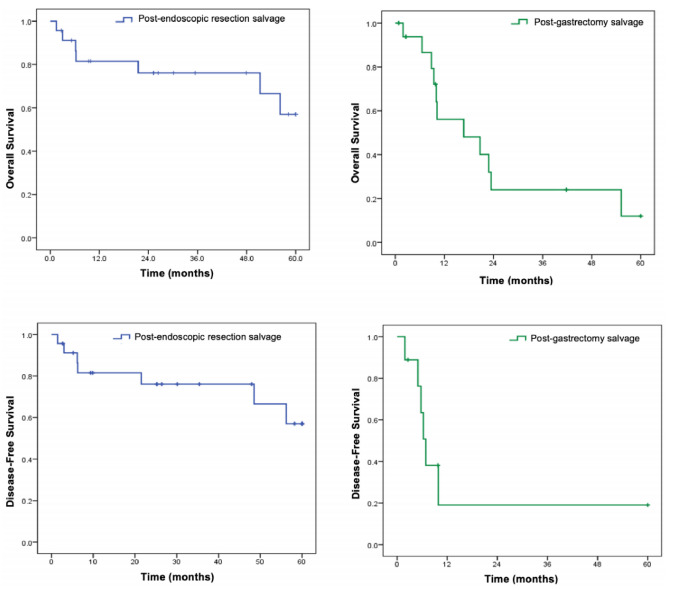
Survival of patients undergoing salvage surgery according to the initial treatment performed.

Regarding OS, patients who underwent SS after endoscopic resection had a mean OS of 46 months, whereas this was 24 months following gastrectomy. The mean DFS was 46 and 16.5 months, respectively. Survival is presented in Figure 1.

## DISCUSSION

Salvage surgery is considered a second chance for cure in cases of unsuccess or recurrence after definitive treatment. Literature about SS in GC is scarce and it mostly comes from small case series. The term is often used as a synonym for palliative or conversion surgery.^6^ ^,^ ^27^ Palliative surgery intends to relieve symptoms without the possibility or intention to cure. In contrast, conversion surgery is performed when an initially incurable patient became potentially curable after chemotherapy or chemoradiotherapy.^9^ ^,^ ^17^

Currently, there is no curative treatment for GC that does not involve resection.^21^ In our institution, complete response is observed in only 5% of those who underwent neoadjuvant therapy.^16^ Patients with lymphoma with disease persistence restricted to the stomach following treatment are an exception.^24^ Therefore, as mentioned earlier, there are three possible scenarios for SS in GC.

The most common scenario is SS after endoscopic resection. This is accepted as a curative treatment for early GC when the risk of lymph node metastasis is negligible.^8^ ^,^ ^12^ In order to be considered curative, endoscopic resection must meet all the classic criteria recommended by the JGCA.^12^ It is controversial whether the expanded criteria also apply to Western patients.^18^ Noncurative endoscopic resection (final pathological report with noncurative factors) is associated with a risk of local recurrence of 2.0-35.1%^5^ and, when followed by SS, 5.0-13.0% had a residual tumor, and 4.3-13.4% had lymph node metastasis.^13^ Considering this, gastrectomy with lymphadenectomy may be recommended in Brazil, when the lesion extrapolates the traditional criteria and in those with disease relapse. In this study, 87% of the indicated cases had a residual tumor and, in 17.3% cases, lymph node metastasis was observed. Nonetheless, there is no consensus on the indication for SS after endoscopic resection that goes beyond the traditional or even the expanded criteria.

Hatta et al.^7^ conducted a retrospective multicenter study evaluating 2,006 patients, in which 1,101 patients underwent salvage gastrectomy and 905 patients were exclusively followed. The patients were stratified by clinicopathological characteristics, according to the risk of lymph node metastasis and disease-specific survival (DSS), creating the eCura score. Patients classified as low risk had a DSS of 99.6% in 5 years and only 2.5% of lymph node metastasis, indicating that SS may be avoided in this subgroup. Niwa et al^14^ applied the eCura score to 47 patients undergoing SS and did not find any remaining disease in those classified as low risk. Even though the sample was small, those who classified as high risk benefited from the salvage.^7^ ^,^ ^14^ Kim et al.^10^ compared 194 patients undergoing SS with 80 patients who were followed only clinically. A greater survival was noticed for the operated ones. Another study showed that when there is recurrence after noncurative ESD, survival is poor even when SS is performed.^25^ In a meta-analysis with 4,780 patients after noncurative endoscopic resection, the OS and DFS at 5 years were better in those who underwent SS. This was also observed in those above 75 years of age. These results must be considered in the context that selection bias might occur and only those patients with good clinical performance received SS. In addition, rescue gastrectomy was not compared with other treatment modalities, such as endoscopic resection and endoscopic ablation.

It is worth mentioning that in our series, salvage gastrectomy was curative in all cases. Major complications were acceptable (8.7%) and, interestingly, the average LOS was long (15.7 days). There was one relapse, which was expected since the advanced cases are included in the cohort.

When it comes to SS, the second scenario is the most commonly acknowledged. In fact, regional relapse is usually systemic, and SS is rarely indicated. The procedure is technically demanding; in nearly half of the times, it is aborted; and multivisceral resection is commonly required (45-92%).^1^ ^,^ ^2^ ^,^ ^15^ ^,^ ^20^ ^,^ ^22^ There are only small series currently available in the literature (Table 4). In our institution, exclusive regional recurrence occurred in 52 (7.3%) of 707 patients undergoing radical surgery. Of these, 16 patients were indicated for SS (23% of exclusive regional recurrence) and, in only 8 (50%) patients, curative resection was obtained. Multivisceral resections were required in 37.5% of these eight patients. Exclusively diagnostic laparoscopy/laparotomy was performed in four patients, and noncurative surgery (bypass or debulking) was performed in another four patients. We also referred four patients from other institutions for SS.

**Table 4 -:** SS in regional recurrence.

Author	Salvage surgery		Complications	Survival
	Performed	Indicated		
*Shchepotin,1995* ^20^	75	40	15%	20% (2y); 66% (salvage + CMT)
*Nunobe, 2011* ^15^	-	36	36%	36% (3y), 10%(5y)
*Badgwell, 2009* ^1^	60	29	52%	38% (3y) e 28% (5y)
*Yoo, 2000* ^28^	97	19	-	22m (median)
*Kodera, 2003* ^11^	-	15	-	38 m (median)
*Sunagawa, 1984* ^22^	-	13	7.6%*	41% (1y)
*Carboni, 2005* ^2^	13	6	33%	13m (median)
*Present study*	16	8	33%	24m (mean)

Y, years; m, months; *mortality; CMT, chemotherapy.

In this scenario, resection with free margins correlates with longer survival.^1^ ^,^ ^2^ ^,^ ^11^ Nunobe et al.^15^ achieved R0 resection in 29 (80.5%) of 36 patients, with greater survival in the R0 group (33 months vs. 6 months). The median survival of the cohort was 23 months, while the DFS in those resected with free margins was 12.5 months (median). Seven patients were survived more than 3 years. However, possible biases are worth mentioning, such as the small number of patients included, the lack of a control group with patients exposed to nonoperative treatment, the inclusion of five patients with peritoneal recurrence, and only bypass was performed in one patient. In our series, as the number of cases is too small, R0 versus R+ was not compared.

Badgwell et al.^1^ performed salvage gastrectomy in 29 out of 60 indicated patients. Patients in whom the initial surgery was not radical (inadequate lymphadenectomy with <16 lymph nodes) and others with metastatic implants in the surgical wound (2 patients) were included. Median survival was higher in the resected group (25.8 months vs. 6 months).

In the largest series available, 75 rescue attempts were performed, with a success rate of 53.3%. The median survival rates of patients undergoing bypass or exclusive laparotomy were 3.1 and 4.5 months, respectively. In resected patients, the 2-year survival was 20% exclusively with surgery, 31% with surgery plus radiotherapy, and 66% with surgery plus chemotherapy.^20^ These findings indicate the need for multimodal treatment.

Although SS for recurrence carries a high risk of complications and high mortality (3-17%), when resection is obtained, it increases survival and might be the only chance for cure. In the assessed cohort, the group indicated for salvage after curative gastrectomy had a mean survival of 24 months and a mean DFS of 16.5 months. It is important to highlight that even after resection, recurrence is high, and OS is poor.

Finally, there is the possibility of surgically rescuing patients who received gastrectomy for cancer, according to the recommendations of the JGCA, but had the residual microscopic disease.^12^ If the lymphadenectomy was inadequate with gross residual disease, or if the initial diagnosis was benign disease and the final pathological examination revealed an adenocarcinoma, surgery may even be recommended, but it cannot be considered salvage by definition. Chen et al.^3^ selected 122 patients with R1 resection who underwent SS. It was possible to obtain free margins in 50 (41%) of them. Survival was significantly better when compared with 72 patients with a second noncurative resection (23 months vs. 18 months). The authors also noted that pN3 patients did not benefit from the second surgical approach, despite being R0.

This study has some limitations. The series is small and patients undergoing salvage were not compared with those who were clinically followed or exclusively underwent chemotherapy (with or without radiotherapy). Furthermore, this is a retrospective evaluation. Despite all this, and as far as we know, it is the first Brazilian study to demonstrate the results of SS in GC and our data are comparable with the findings of other authors, demonstrating its external validation.

## CONCLUSION

Salvage surgery offers the possibility of disease control and increased survival rate in selected patients. The success rate of SS is high after noncurative endoscopic resection. For regional recurrence, salvage surgery is rarely indicated and has a considerable chance for unsuccess, significant morbidity, but is also the only chance for cure.
